# Development and characterization of single domain monoclonal antibody against programmed cell death ligand-1; as a cancer inhibitor candidate

**DOI:** 10.22038/IJBMS.2022.62522.13834

**Published:** 2022-03

**Authors:** Akbar Oghalaie, Fereidoun Mahboudi, Fatemeh Rahimi-Jamnani, Somayeh Piri-Gavgani, Fatemeh Kazemi-Lomedasht, Ayda Hassanzadeh Eskafi, Delavar Shahbazzadeh, Ahmad Adeli, Yeganeh Talebkhan, Mahdi Behdani

**Affiliations:** 1 Venom and Biotherapeutics Molecules Laboratory, Biotechnology Research Center, Pasteur Institute of Iran, Tehran, Iran; 2 Biotechnology Research Center, Pasteur Institute of Iran, Tehran, Iran; 3 Department of Mycobacteriology and Pulmonary Research, Pasteur Institute of Iran, Tehran, Iran; 4 Zoonoses Research Center, Pasteur Institute of Iran, Amol, Iran

**Keywords:** Cancer, Checkpoint inhibitors, Nanobody, Programmed cell death - ligand-1, Single domain antibody

## Abstract

**Objective(s):**

One of the important interactions in controlling the human immune system is the reaction between checkpoint proteins such as programmed cell death-1 (PD-1) and its ligand, PD-L1. These are negative immunoregulatory molecules that promote immune evasion of tumor cells. PD-L1 expression is an immune-mediated mechanism used by various malignant cells in order to down-regulate the immune system. Checkpoint inhibitors (CPIs) are a new class of anti-cancer agents that stimulate immune cells to elicit an antitumor response by blocking the ligand and receptor interactions. Nanobody (Nb) as a new type of antibody fragment, has some potential as CPI.

**Materials and Methods:**

A female camel was immunized with recombinant PD-L1 protein, nanobody library was constructed and PD-L1 specific Nb was selected. The selected Nb was characterized in terms of affinity, specificity, and binding potency in ELISA, Western blotting, and flow cytometry.

**Results:**

Developed nanobody, A22 binds to its cognate target with high specificity and affinity. Western blot and flow cytometry techniques showed that nanobody A22 was able to specifically detect and attach to human PD-L1 protein on the cell surface and in the cell lysate. MTT assay showed the inhibitory effect of PD-L1 by specific Nb on A431 and HEK293 cells, with no cytotoxic effect on cell growth.

**Conclusion:**

The results highlighted the potential of anti-PD-L1 Nb as a novel therapeutic in cancer therapy without undesirable cytotoxicity.

## Introduction

One of the important functions of the immune system is the ability to distinguish between normal and abnormal cells in the body by checkpoint proteins. Immune checkpoints are certain molecules on specific immune cells that should be activated (or inactivated) to start an immune response (1, 2). These immuno-regulatory agents act by limiting the T cell activity and developing self-tolerance. The well-characterized checkpoint proteins are programmed cell death-1 (PD-1), programmed cell death ligand-1 (PD-L1), and cytotoxic T-lymphocyte-associated protein 4 (CTLA-4). These proteins prevent excessive inflammation and act as a “switch off” to prevent T cells from invading normal cells (2). PD-L1 is a human cell surface protein encoded by the *PDCD1* gene located at p24.1.2 position on chromosome 9 which binds to PD-1 protein and has been introduced as the third member of the B7 protein family (3, 4). The intracellular part of PD-L1 consists of a short cytoplasmic tail (30 amino acids) that is responsible for signal transduction (3, 4). PD-L1 is normally expressed by CD8^+^ T cells and leads to inhibition of TCR signaling via the SHP1/2 pathway (5, 6). The binding of T-cell-associated PD-1 protein to its ligand which is located on macrophages, dendritic cells, and tumor cells transmits signals and reduces the activity of cytotoxic T cells. In chronic immune responses and tumors, interferon-gamma (IFN-γ) produced by T cells induces the expression of PD-L1 at the antigen-presenting cells and tumor cells followed by down-regulation of the immune responses, which eventually yield to the failure of immunostimulants (7, 8). This reduced anti-tumor immune response usually occurs in two ways: i) inactivation of cytotoxic T cells in the tumor microenvironment (5, 6) and ii) inhibition of new cytotoxic T cell activation within the lymph nodes (9-11). A high expression level of PD-L1 allows cancer cells to “trick” the immune system and prevent attacks as foreign harmful substances. Previous studies have shown that high expression of PD-L1 in tumor cells increases the risk of death by increasing tumor invasion (12). Checkpoint inhibitors (CPIs) are a new class of anti-cancer agents that stimulate immune cells to elicit an anti-tumor response by blocking the ligand and receptor interactions. Antibodies have been considered as CPI as well (1). 

Heavy chain antibodies (HCAbs; ~ 95kDa), introduced by Hamers-Casterman *et al*. (13), are naturally devoid of light chains and therefore recognize their cognate antigens by a single variable-domain, referred to as VHH or Nanobody (Nb). Their size is approximately 2.5 nm in diameter and 4 nm long and weigh about 12-15kDa (13-15). The biochemical and pharmacokinetic properties of Nbs make these small molecules ideal tools for targeted drug delivery, immunotherapy, and medical diagnosis (16, 17). Considering the important role of the PD-1/PD-L1 molecular pathway in the development of various types of cancer, in this study we characterized PD-L1 specific Nb from immunized camel Nb, and the binding and functionality of the achieved Nb was evaluated using *in vitro* assays.

## Materials and Methods

***Immunization procedure***

A six-month female *Camelus dromedarius* was immunized subcutaneously (*s.c*) with 200 µg of the previously developed recombinant extracellular domain of PD-L1 produced in *Escherichia coli *six times at one-week intervals. Freund’s complete and incomplete adjuvants were used for the first and booster injections, respectively. Blood samples were taken after each injection and serum was isolated. The immune responses were analyzed by enzyme-linked immunosorbent assay (ELISA). Briefly, 1 µg/ml of recombinant PD-L1 was coated in a Maxisorp 96-well plate overnight at 4 °C. The wells were blocked with phosphate-buffered saline (PBS) supplemented with 2%w/v skim milk and incubated at room temperature (RT) for 1 hr. Serially diluted sera were added to the wells and incubated for 1 hr. The wells were washed 5 times with PBS-Tween20 (0.05%) (PBST) and then, rabbit anti-camel antibody (1:2000 dilution) was added to the wells and incubated for 1 hr (22). After the washing step, goat anti-rabbit IgG antibody (HRP) (1:2000 dilution) was added and incubated for 1 hr. The wells were washed again and 3,3’,5,5’-Tetramethylbenzidine (TMB) was added to the wells and incubated for 15 min in the dark. The reaction was stopped with 2N H_2_SO_4_ and optical density (OD) was measured at 450 nm.

***Immune library construction***

The peripheral blood mononuclear cells (PBMCs) were collected using ready-to-use Ficoll density gradient media (Sigma, USA). Total RNA was purified using an RNA extraction reagent and then cDNA was synthesized using oligo dT primers. VHH fragments were amplified using nested primers. The first amplification round was carried out with CALL001 (5’-gtcctggctgctcttctacaagg-3’) and CALL002 (5’-ggtacgtgctgttgaactgttcc-3’) primers corresponding to leader sequence and CH2 domain of HcAb. The amplified fragments (600–700bp) were extracted from agarose gel and used for the second nested PCR with A6E (5’-gatgtgcagctgcaggagtctggtggagg-3’) and P38 (5’-ggactagtgcggccgctggagacggtgacctgggt-3’) primers containing *Pst*I and *Not*I restriction sites, respectively. The PCR product (~ 400 bp) and pHEN-4 phage display phagemid were digested, gel purified, and ligated using T4 DNA ligase. The recombinant phagemid (pHEN-4-VHH) was electroporated into *E. coli* TG1 competent cells and cultured on LB agar plates supplemented with appropriate antibiotic (ampicillin). Approximately, 1×10^12^ colony forming units (CFUs) of VCSM13 helper phage were added to the TG1 cells (at logarithmic phase, OD_600_ of 0.4–0.6) and incubated at 37 °C without shaking. After 30 min, kanamycin was added to the culture medium and incubated overnight at 37 °C while shaking at 250 rpm. The bacterial pellet was collected by centrifugation at 8000×g for 10 min. Recombinant phages were purified from the supernatant of the culture medium using PEG-NaCl solution (20 % PEG 6000, 2.5 M NaCl) after one hour of incubation on ice. The phage library was collected using centrifugation at 10,000×g for 15 min. 

***Biopanning***

Phages displaying PD-L1 specific Nbs were enriched using biopanning. Four successive rounds of biopanning were performed on immobilized PD-L1. Briefly, a 96-well plate (NUNC, Denmark) was coated overnight at 4 °C with 10 µg/ml of PD-L1 in sodium bicarbonate buffer (pH 9.6). The negative control wells were coated with 100 µl of the buffer alone. The wells were blocked with 2% skim milk in PBS for 1 hr at RT. About 1×10^11^ CFU of phage library was added to the wells and incubated for 1 hr (RT). The wells were washed 10 times with PBST and bounded phages were eluted with 100 mM triethylamine (pH 10.0). Then, 100 µl of 1M Tris-HCl (pH 8.0) was added as the neutralizer. Ten-fold serial dilution of eluted phages (output phages) was prepared and used for infection of TG1 cells (log phase OD_600_ 0.4-0.6). The dilution series was cultured on 2×YT agar plates containing ampicillin. The remaining output phages were amplified in TG1 cells and rescued by VCSM13 helper phage for a subsequent round of biopanning. All steps of consecutive rounds of biopanning were identical except for the washing step in the increased concentration of tween-20 from 0.05 to 0.5%v/v (0.05, 0.1, 0.2, and 0.5 %) to increase the stringency of the biopanning procedure and obtain PD-L1 bounded phages with higher specificity and affinity. 

***Biopanning monitoring by polyclonal phage ELISA***

To evaluate the outcome of the biopanning process, polyclonal phage ELISA was performed using output phages. Briefly, a 96-well plate was coated with 1 µg/ml of PD-L1 overnight at 4 °C. After blocking the wells with 2% skimmed milk, 1×10^10^ CFU of output phages were added to the wells and incubated for 1 hr (RT). The wells were washed 10 times with PBST, and anti-M13 HRP-conjugated antibody (1:2000) was added and incubated for 1 hr. Then, the wells were washed, and an ELISA reaction was developed using TMB followed by 2N H_2_SO_4_. The optical densities were measured at 450 nm.

***Screening of the nanobody library***

Over 19 individual colonies from the fourth round of biopanning were randomly picked up and cultured. Expression of periplasmic Nb-PIII fusion protein was induced with 1 mM isopropyl d-1-thiogalactopyranoside (IPTG) and used for PD-L1 detection in ELISA. ELISA was performed as previously described according to the polyclonal phage ELISA to analyze the differences of individual colonies in antigen recognition. After coating and blocking steps, periplasmic extracts (PE) were added to the wells and the presence of Nbs was recognized using anti-HA antibody (1:2000) followed by anti-mouse HRP-conjugated antibody (1:5000). Colonies representing optical densities three times over the control samples were considered as positive and submitted for sequencing analysis.

***Expression and purification of selected nanobody***

After identification of positive clones through PE-ELISA, the sequence analysis was performed and the results were blasted in NCBI and numbered using the IMGT database. Selected vectors were extracted and used as the template for Nb gene amplification with A6E and P38 primers. PCR product was gel extracted and digested with *BstE*II and *Pst*I restriction enzymes and ligated into pHEN6c plasmid. The recombinant plasmid (pHEN6c-A22) was transformed into *E. coli* WK6 cells using heat shock and CaCl_2_ (1). The expression of recombinant nanobody was induced with 1 mM IPTG at 28 °C overnight. Periplasmic fraction of the WK6 cells was extracted by osmotic shock and Nb was purified using Ni-NTA chromatography according to the manufacturer’s instructions. Purified Nb was dialyzed against PBS and concentrated using a Vivaspin concentrator (Cutoff: 10kDa). The proteins were analyzed on 15% sodium dodecyl sulfate-polyacrylamide gel electrophoresis (SDS-PAGE) and by coomassie brilliant blue staining. For western blotting, protein bands were transferred onto the nitrocellulose membrane. The membrane was then blocked with 4% skim milk for 2 hr (RT). Then, an anti-Histidine HRP-conjugated antibody (1:2000) was added and incubated for 4 hr. The protein bands were visualized using 3,3’-Diaminobenzidine (DAB) chromogenic substrate (13, 18). 

***Affinity analysis***

The binding constant of Nb was evaluated using Beatty’s protocol as described previously (19). A checkerboard assay with serial dilution of PD-L1 as well as Nbs was performed to achieve a saturating concentration of PD-L1 and Nbs. Two different concentrations of PD-L1 (0.1 and 1 µg/ml) were coated at 4 °C overnight. Various concentrations of Nbs (0 to 10 nM) were added and incubated at RT for 1 hr. Detection of Nb/PD-L1 binding was evaluated by anti-His HRP-conjugated antibody (1:2000). The sigmoid curve of Nb against PD-L1 antigen was drawn and K_aff_ of each Nb was calculated using the following equation: 

N = [Ag] / [Ag’]

K_aff_= N – ½(N[Nb] – [Nb])

Where [Ag] and [Ag’] refer to 1 and 0.1 µg/ml concentrations of PD-L1, [Nb], and [Nb’] refer to the concentrations of Nb at the maximum half binding of [Ag] and [Ag’], respectively.

***Binding analysis***

The binding capacity of the soluble Nb to PD-L1 and other antigens such as programmed cell death protein 1 (PD-1), vascular endothelial growth factor (VEGF), vascular endothelial growth factor receptor 2 (VEGFR2), neuropilin-1 (NRP-1), epithelial cell adhesion molecule (EpCAM), zinc transporter protein (LIV-1), cytotoxic T-lymphocyte–associated antigen 4 (CTLA-4), bovine serum albumin (BSA), and casein was evaluated in solid ELISA experiments. 

***Flow cytometry***

A431 and HEK293 cell lines were used for flow cytometry analysis. 3×10^5 ^cells were counted and washed three times with PBS (1% BSA) and incubated with 1 µg of PD-L1 specific Nb at 4 °C for 1 hr (up to 100 µl). The cells were washed and incubated with 1 µg of rabbit anti-His antibody. After washing steps, 1 µg anti rabbit FITC conjugated was added to the wells and incubated at the same condition for 1 hr. The cells were washed and monitored using Cyflow (Partec, Sysmex) (20).

***Cytotoxicity assay***

Cytotoxicity of the recombinant Nbs was evaluated by 3-(4,5-dimethylthiazol-2-yl)-2,5-diphenyltetrazolium bromide (MTT) assay. 3×10^5^ of A431 and HEK 293 cells were cultured in 96-well sterile plate in DMEM medium supplemented with 10% FBS. Serial dilution of Nbs (0 to 100 nM) was added to the wells and incubated for 24, 48, and 72 hr at 37 °C, under 5% CO_2_. PBS was added to the control wells and incubated at the same condition. 50 µl of ready-to-use MTT solution (5 mg/ml) was added to the wells and incubation was carried out for a further 4 hr in the dark. MTT solution was removed and formazan crystals were solubilized with 100 µl of dimethylsulfoxide (DMSO). The OD was measured at 570 nm (630 nm as a reference wavelength).

## Results

***Monitoring of camel immunization procedure***

**Figure 1:**
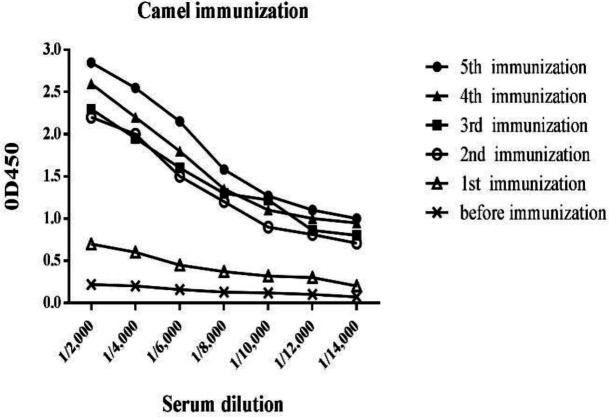
Antibody titration against recombinant PD-L1 after camel immunization procedure

The camel was immunized six times with recombinant extracellular domain of PD-L1 and the immunization process was analyzed using ELISA. As shown in Figure 1, the antibody titer was raised after the second injection which indicated the successful immunization process.

***Library construction and biopanning***

**Figure 2:**
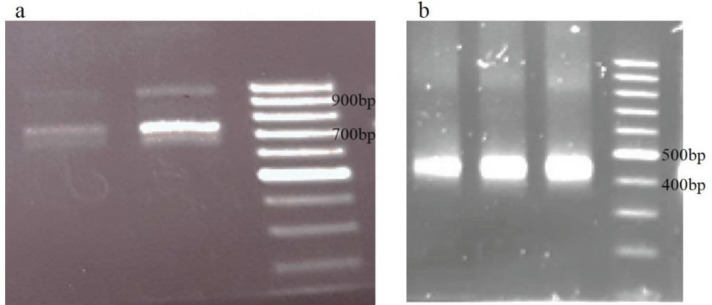
VHH gene amplification. Amplification of VH and VHH genes by the 1st PCR (a); the second nested PCR for VHH gene (b)

cDNA was synthesized from RNA samples extracted from PBMCs and used for amplification of gene fragments encoding the variable domains of the heavy-chain antibodies. The 1^st^ PCR amplified two distinct PCR products (Figure 2a) while the 2^nd^ PCR amplified the VHH sequences (approximately 400 bp) (Figure 2b). The PCR products were ligated into pHEN4 phagemid and transformed into *E. coli* TG1 cells. Following transformation, a library of about 4×10^7^ transformed bacteria was obtained in which transformation efficiency was 90% through colony-PCR.

After each round of biopanning, the library enrichment was qualitatively investigated. In this way, the bounded phages in both positive (antigen-coated wells) and negative (wells without antigen) wells were eluted and used to infect TG-1 cells. After 16 hr incubation, the number of colonies grown in each dilution step was counted and divided by the number of colonies grown in the corresponding negative well, and the degree of enrichment was calculated. Table 1 shows appropriate results of the enrichment process. 

***Polyclonal phage ELISA***

**Figure 3:**
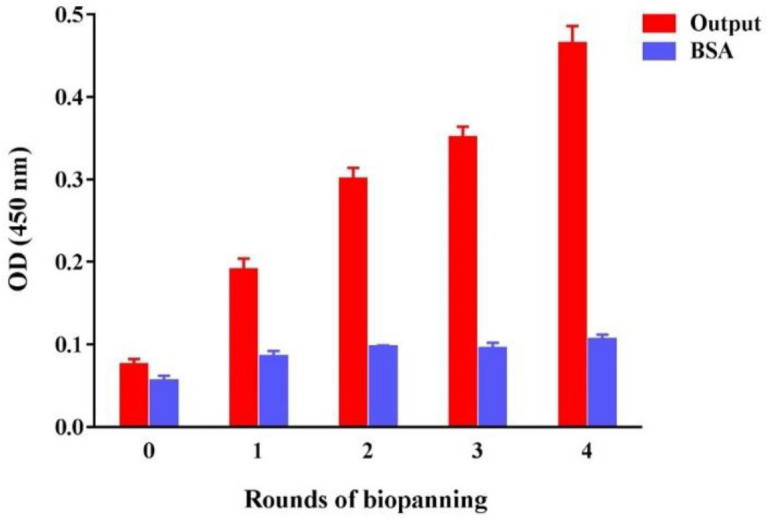
Polyclonal phage-ELISA for evaluation of the biopanning procedure

The phages obtained after each round of biopanning (output phages) were evaluated by an ELISA assay. It was expected that by increasing the rounds of enrichment, higher optical densities would be obtained due to the increased number of specific phages against the coated antigen. The results showed an upward trend indicating a successful library enrichment procedure (Figure 3). 

***Specific nanobody selection***

**Figure 4:**
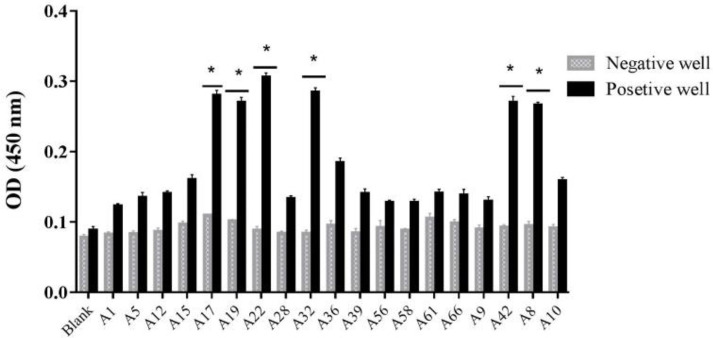
PE-ELISA assay for selection of individual nanobodies

After the 4^th^ round of biopanning, 19 single colonies were selected and screened by PE-ELISA. Six colonies showed positive signals which were at least 3 times higher than negative controls (Figure 4). The colony representing the highest optical density (#A22) was selected and its nucleotide sequence was identified.

***Expression and purification of anti-PD-L1 nanobody***

**Figure 5:**
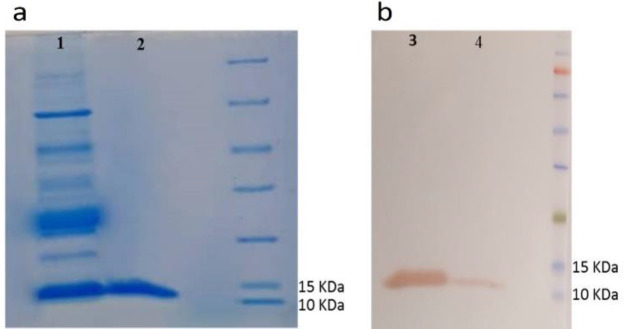
Protein expression; SDS-PAGE (a) and Western blotting (b) of Nb. A22. #1, 4: Lysate of recombinant clones after induction; #2, 3: Purified Nb. A22 (13kDa)

After subcloning of gene fragment encoding Nb. A22 into pHEN6c expression vector, protein expression was performed in TB medium and the Nb was extracted from the periplasmic space by osmotic shock. The yield of protein expression (2 mg/l) was calculated by the adsorption rate at 280 nm. The purified Nb was evaluated on 15% SDS-PAGE (Figure 5a) and its identity was confirmed using western blotting with anti-His antibody (Figure 5b). 

***Affinity and specificity analysis***

**Figure 6:**
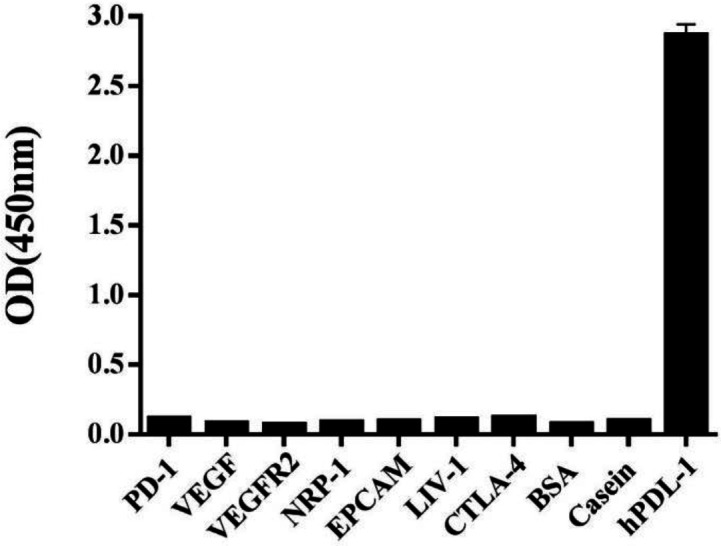
Specificity analysis of Nb. A22 by ELISA

The affinity of Nb. A22 was evaluated according to Beatty’s protocol (19). First, adsorption of different concentrations of Nb in two concentrations of antigen was calculated. Then, using Beatty’s affinity determination formula, the affinity of Nb was calculated which was 7×10^12^ M^-1^. Binding capacity and cross-reactivity of Nb. A22 with PD-L1 as well as other antigens were tested by ELISA. The results showed that PD-L1 specific Nbs could not react with other antigens including PD-1, VEGF, VEGFR2, NRP-1, Ep-CAM, LIV-1, CTLA-4, BSA, and casein (Figure 6).

***Flow cytometry analysis***

**Figure 7:**
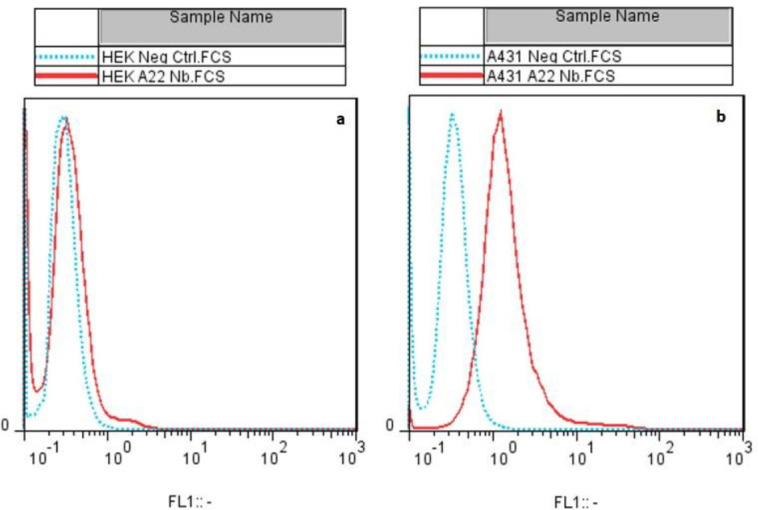
Binding analysis of nanobody. A22 by flow cytometry; HEK293 (PD-L1 negative) (a) and A431 (PD-L1 positive) (b) cell lines. Solid and dotted histograms indicated cells treated with and without Nb. A22, respectively

To verify the binding capacity of PD-L1 specific Nb in the detection of the native form of PD-L1 on the surface of A431 cells, flow cytometry analysis was performed. The results indicated strong binding of Nb. A22 to A431 cells (PD-L1 positive cell line). However, no binding of Nb to HEK293 cells (PD-L1 negative cell line) was observed (Figure 7).

***MTT assay***

**Figure 8:**
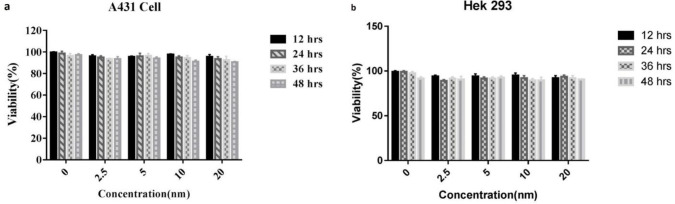
Cytotoxicity assay of A431 (a) and HEK293 (b) cells treated with nanobody (Nb)

The inhibitory effect of PD-L1 specific Nb on A431 and HEK293 cells was tested by MTT assay. It was shown that Nb. A22 did not have any effect on cellular viability (Figure 8). 

**Table 1:** Semi-quantitative analysis of biopanning procedure

Biopanning rounds	Number of colonies in hPD-L1 coated wells (CFU) (A)	Number of colonies in negative wells (CFU) (B)	Enrichment ratio (A/B)
1	205	110	1.86
2	190	95	2
3	180	30	6
4	605	29	20.8

## Discussion

Monoclonal antibodies (mAbs) can be produced by several approaches including hybridoma technology, repertoire cloning, CRISPR/Cas9, phage, and yeast display technologies (21), which can be applied to enhance the specificity, stability, therapeutic efficacy, and capacity. Compared with the polyclonal antibodies which can bind to several epitopes, mAbs are high-specific universal binding molecules. These specific molecules are essential tools in research, diagnosis, and treatment. Therapeutic mAbs are divided into four groups: Murine, Chimeric, Humanized, and Human. Currently, mouse antibodies are not used due to immunological reactions, and fully human antibodies are the most desirable therapeutic molecules due to their origin and lack of side reactions. Among therapeutic mAbs, the most effective drugs include adalimumab (Humira), an mAb used to treat rheumatoid arthritis, and bevacizumab (Avastin) which targets vascular endothelial growth factor (VEGF) and inhibits the growth of blood vessels (19). One of the most important disadvantages of human therapeutic antibodies is the need for mammalian expression systems which increase the cost of production. An alternative approach is developing nanobodies which have attracted much attention due to their small size which ensures their improved tumor penetration, rapid diffusion, fast clearance from the body, binding to hidden epitopes, low immunogenicity, safety for humans, and low cost of production.

Many studies have been conducted or are being done on development of Nbs against different targets in research, preclinical and clinical stages (22). Nowadays, one of the nanobodies that has received the European Medicines Agency (EMA) and the USA Food and Drug Administration (FDA) approval is Caplacizumab which has been designed for treatment of thrombotic thrombocytopenic purpura by targeting the von Willebrand Factor (vWF) (23). The development of nanobodies against tumor antigens is growing rapidly and various targets have been considered. Human epidermal growth factor receptor 2 (HER2) is one of the tumor markers for Nb development. One of the first attempts in the development of anti-HER2 Nbs was conducted by Vaneycken *et al*. (24) in which a panel of 38 anti-HER2 Nbs was biochemically characterized and preclinically evaluated for utilization as tracer for imaging of xenografted tumors (25-29). These nanobodies have a low half-life which causes a rapid clearance of radioactive or other toxic substances. Another application of tumor-specific nanobodies is targeting the tumor-associated ligands such as VEGF and placental growth factor (PlGF). In the study conducted by Kazemi *et al*., an anti-VEGF Nb was developed which had high specificity and binding affinity towards both human and mouse VEGF. This Nb potently inhibited human endothelial cell migration and tumor growth in a tumor-bearing mouse model (30). 

In the current study, we developed a human PD-L1 specific Nb by phage display technique. Four consecutive rounds of biopanning were performed to obtain Nbs with the highest affinity and specificity to immobilized PD-L1. The output of the biopanning rounds was checked through polyclonal ELISA which represented a successful Nb library enrichment procedure. Screening of Nb library through PE-ELISA on selected clones yielded the highest optical density in ELISA. The advantage of PE-monoclonal ELISA over monoclonal phage ELISA is that in the former binding capacity of soluble Nb (fused to phage protein III) to the immobilized antigen will be evaluated and the results will be more reliable. Selected Nb was subcloned in pHEN6c expression vector expressed within WK6 cells. Recombinant Nb. A22 was purified in the native structural form from the periplasmic space by osmotic shock. The calculated affinity of the selected Nb was in the range of picomolar which was comparable with previously reported studies (16, 31). The results of flow cytometry analysis indicated strong binding capacity of Nb. A22 to PD-L1 antigen presented on A431 cell line in comparison with PD-L1 negative HEK293 cells. The MTT results showed no cytotoxicity effect of anti-PD-L1 Nanobody on A431 and HEK293 cells. A similar study, developed a nanobody library of PD-L1 immunized camel and obtained 3 anti-PD-L1 nanobodies that had amino acid differences in the CDR regions and their affinity was reported in the range of nanomolar (32). In another study, Zhang *et al*. developed an anti-PD-L1 Nb which is a potent inhibitor of the PD-1/PD-L1 interaction with strong antitumor activity within a mouse model (33). 

## Conclusion

Taken together, in the present study Nb with a high and reasonable affinity and specificity was obtained using phage display technology from the Nb library developed by PD-L1 immunization of a camel. Selected Nb showed specific binding in ELISA and flow cytometry assay and did not have any cytotoxicity effect on treated cells. The results indicated the potential role of anti-PD-L1 Nb as a promising tool in cancer therapy. The anti-tumor activity of the selected Nb should be further evaluated in *in vivo *experiments before making any firmed conclusion. 

## Authors’ Contributions

AO, MB, FK, and FM Conceived the study and design; FM, F, and FRJ Analyzed the data and prepared the draft manuscript; MB and YT Critically revised the paper; DS and AA Supervised the research; SPG and AHS Processed and collected data and performed experiments.

## Conflicts of Interest

The authors have no conflicts of interest. 
